# Two Novel lncRNAs Regulate Primordial Germ Cell Development in Zebrafish

**DOI:** 10.3390/cells12040672

**Published:** 2023-02-20

**Authors:** Wenjing Li, Wei Liu, Chengyu Mo, Meisheng Yi, Jianfang Gui

**Affiliations:** 1 School of Marine Sciences, Sun Yat-sen University, Zhuhai 519082, China; 2 Southern Marine Science and Engineering Guangdong Laboratory, Zhuhai 519082, China; 3 Guangdong Provincial Key Laboratory of Marine Resources and Coastal Engineering, Guangzhou 510275, China; 4 State Key Laboratory of Freshwater Ecology and Biotechnology, Institute of Hydrobiology, The Innovation Academy of Seed Design, Chinese Academy of Sciences, Wuhan 420072, China

**Keywords:** lncRNA, primordial germ cell, cell migration, zebrafish

## Abstract

Long noncoding RNAs (lncRNAs) are regulatory transcripts in various biological processes. However, the role of lncRNAs in germline development remains poorly understood, especially for fish primordial germ cell (PGC) development. In this study, the lncRNA profile of zebrafish PGC was revealed by single cell RNA-sequencing and bioinformatic prediction. We established the regulation network of lncRNA-mRNA associated with PGC development, from which we identified three novel lncRNAs—*lnc172*, *lnc196*, and *lnc304*—highly expressing in PGCs and gonads. Fluorescent in situ hybridization indicated germline-specific localization of *lnc196* and *lnc304* in the cytoplasm and nucleus of spermatogonia, spermatocyte, and occyte, and they were co-localized with vasa in the cytoplasm of the spermatogonia. By contrast, *lnc172* was localized in the cytoplasm of male germline, myoid cells and ovarian somatic cells. Loss- and gain-of-function experiments demonstrated that knockdown and PGC-specific overexpression of *lnc304* as well as universal overexpression of *lnc172* significantly disrupted PGC development. In summary, the present study revealed the lncRNA profile of zebrafish PGC and identified two novel lncRNAs associated with PGC development, providing new insights for understanding the regulatory mechanism of PGC development.

## 1. Introduction

Primordial germ cells (PGCs), the precursors of germline, segregate at early embryonic development and migrate towards the genital ridge, where they proliferate and differentiate into gonocytes that give rise to gametes [1,2,3]. The migration process imposes significant challenges for PGCs, as along the migratory route PGCs are exposed to various signals that direct differentiation of somatic cell types [4,5,6]. In *Drosophila melanogaster*, PGCs migrate across the midgut to the mesoderm towards the somatic gonadal precursors [7]. In addition, mouse PGCs migrate from the posterior primitive streak over the endoderm, across the midgut to reach mesoderm, where the primordial gonad develops [8]. Fish PGCs find their way to the genital ridge by a complex migration route through six distinct steps using intermediate targets throughout the embryo [9]. The chemokine-guided cell migration is one of the most important principles of directing PGC migration, conserved in fish and mammals [10]. The G protein–coupled receptor (GPCR)–mediated chemokine attractant is essential for guiding PGC migration towards the genital ridge, such as that trapped in endoderm 1 (TRE1) in *Drosophila melanogaster* [11] and chemokine (CXC motif) receptor 4b (CXCR4B) in teleost and mammals [10,12]. 

In lower animals, including teleost fish, PGC development is dependent on molecules that reside within the maternally inherited germ plasm, the non-membrane phase-separated organelle condensates made of RNAs and proteins (RNPs) [13,14,15], which play essential roles in the regulation of germ plasm aggregation, transcriptional repression, fate maintenance, polarity, and motility in PGC [16,17,18,19,20,21,22,23]. In addition to these coding genes, non-coding transcripts including piRNAs and miRNAs also contribute to regulate the directional migration, fate maintenance, and transposon silence of PGC [24,25,26,27]. Long non-coding RNAs (lncRNAs), a class of transcripts consisting of over 200 base pairs with limited coding potential, function as important regulators in many biological processes including germline development [28,29]. In *Xenopus*, the Xlsirts, a group of non-translatable repeat transcripts, are indispensable for the translocation of Vg1 and other heterologous RNAs [30,31]. In *Drosophila*, the lncRNA-*pgc* is required for RNA pol II–dependent transcriptional silence in PGCs via blocking the transition from preinitiation to transcriptional elongation [32,33]. In mice, evidence increasingly shows that lncRNAs play important roles in many reproduction processes including the establishment of pregnancy [34], the maintenance of spermatogonial stem cell [35], the post-meiotic gene expression on the Y chromosome, and the control of sperm qualities [36]. However, studies about lncRNA profiles and their functions in fish germline remain very limited. The first obstacle to studying lncRNA profiles of PGC is that the cell number is too small to afford normal RNA sequencing. 

Single-cell RNA sequencing (scRNA-seq) is facilitated to investigate the transcriptome landscape of stem cell homeostasis [37,38,39]. In zebrafish, complex developmental trees of early embryogenesis have been reconstituted by scRNA-seq [40]. A recent study analyzed the transcriptome files of migratory PGCs at three stages in zebrafish [41]. However, it remains unclear how many lncRNAs are involved in zebrafish PGCs and whether lncRNAs are involved in the regulation of PGC development. To answer this question, we performed scRNA-seq to reveal the lncRNA profiles of PGC. From the lncRNA–mRNA regulation network, we identified two novel lncRNAs, *lnc172* and *lnc304*, associated with PGC migration in zebrafish.

## 2. Materials and Methods

### 2.1. Fish Care and Maintenance

*Tg* (*kop*: EGFP-3′UTR-*nanos*) zebrafish of AB genetic background were obtained from the China zebrafish resource center (CZRC, Wuhan, China). Zebrafish (*Danio rerio*) were raised and maintained at 28 °C with 10 h (h) darkness and 14 h light. The embryos were incubated in Ringer’s solution in 28 °C incubator (Xutemp, China, Hangzhou). All the procedures with zebrafish were approved by the Ethics Committee of Sun Yat-sen University.

### 2.2. Isolation of PGCs

All the embryos were collected after natural spawning and staged as previously reported [42]. GFP-labelled PGC were isolated from Tg embryos at 5 hpf. The chorions were removed by 0.25% pronase (Roche, Mannheim, Germany) in an embryonic medium as the zebrafish book described (http://zfin.org/zf_info/zfbook/zfbk.html, accessed on 1 January 2022). The de-chorion embryos were disassociated to a single cell in a deyolking buffer (55 mM NaCl, 1.8 mM KCl and 1.25 mM NaHCO_3_), rinsed twice in a washing buffer (110 mM NaCl, 3.5 mM KCl and 2.7 mM CaCl_2_ dissolved in 10 mM Tris/Cl, pH 8.5), and resuspended in nuclease-free PBS. Single GFP-labelled PGC was collected with a capillary tube under a fluorescence microscope (Zesis, Observer Z1, Gottingen, Germany). The collected single PGC was frozen in each tube containing the lysis buffer for single-cell RNA library construction from Annoroad gene technology Co. Ltd. (Beijing, China). A total of 30 PGCs were collected in 30 tubes. After quality checking, 3 of the 30 samples were used for scRNA-seq independently.

### 2.3. scRNA-seq

The collected cells were directly amplified to construct the cDNA library by the Smart-Seq2 method [22] and were sequenced on the Illumina HiSeq 2500 by Annoroad gene technology Co. Ltd. (Beijing, China). The raw data have been submitted to the NCBI SRA database (PRJNA811725). The raw data were processed with Perl scripts to filter reads with more than 5 adapter-polluted bases, reads with less than 19 accounting for more than 15% and reads with the number of N bases over 5%. Clean reads were mapped to the reference genome danRer10 downloaded from the Ensembl database using Bowtie2 v2.2.3. Gene expression was calculated with fragments per kilobase million mapped fragments (FPKM).

### 2.4. LncRNA Analysis

Known lncRNAs were obtained by blasting to known lncRNA databases (ZFLNC, NONCODE, NCBI, and Ensembl). To obtain putative novel lncRNAs, potential coding transcripts were removed by three types of forecasting software including the coding potential calculator, pfam-scan, and Coding-Non-Coding-Index as previously described [43]. The cis- and trans-target mRNAs of lncRNAs were computationally predicted with the following rules: the potential cis-targets were the protein-coding genes within a 10 k bp window upstream or downstream of the lncRNAs and with a correlation value larger than 0.9; and the trans-targets were predicted on the basis of the mRNA-lncRNA sequence complementarity and the RNA duplex energy. The sequence complementarity was analyzed by BLAST, and the duplex energy was assessed by RNAplex software with the threshold of correlation larger than 0.9 [44]. Gene Ontology enrichment was analyzed using Goatools (https://github.com/tanghaibao/Goatools, accessed on 1 January 2022).

### 2.5. Reverse Transcription and qPCR Analysis

To validate the scRNA transcriptome, strand cDNA libraries of singe PGC were first constructed with a single cell sequence−specific amplification kit (Vazyme, Nanjing, China). The total RNA of adult tissues and embryos were extracted using a TRIzol reagent (Invitrogen, Waltham, MA, USA), and the RNAs were reverse transcribed with GoScript^TM^ reverse transcription mix, random primer (Promega, Madison, WI, USA). qPCR was performed on the Roche Light Cycler 480II (Roche, Rotkreuz, Switzerland). The thermal cycling conditions were as previously described [26]. *ef1a* and *rpl13a* were used as reference genes to calculate gene expression with 2^-ΔΔCt^ method. All the data were represented with mean ± SD from three independent experiments in triplicates. The primers used in this study are listed in Table S1.

### 2.6. Double Color Staining of Immunofluorescence and Fluorescence In Situ Hybridization

The sequence-specific DNA fragments of *lnc196*, *lnc304*, and *lnc172* were amplified with a pair of primers whose reverse primer contained the core sequence of T7 promoter at the 5′ end (primer and lncRNA sequence information in Table S1). Digoxin-labelled antisense RNA probes were synthesized using the DIG-RNA labeling kit (Roche, Mannheim, Germany). The gonads were fixed in 4% PFA at room temperature for 2 h, washed in PBS for 3 × 10 min, and embedded with frozen section compound (Sakura Finetek, Torrance, USA). The samples were sectioned with a thickness of 10 μm. The slides were dried at 37 °C for 30 min, rehydrated, and washed in PBS three times and hybridized with DIG-labeled anti-sense probes at 60 °C for 16 h. After a series of washing steps, the slides were blocked in 2% blocking reagent (Roche, Mannheim, Germany) in MAB buffer (0.1 M Maleic acid and 0.15 M NaCl, pH 7.4) for over 2 h at room temperature and incubated in anti-DIG-POD FAB fragments (Roche, 1:2000 diluted in 1% blocking solution) and anti-vasa antibodies (abcam, ab209710, 1:400) at 4 °C overnight. After washing in PBT and PBS buffer three times, the slides were incubated in TSA™ Plus Fluorescence Systems (Red) (PerkinElmer), FITC-conjugated goat anti-rabbit IgG (H + L), and DAPI (1 μg/mL) for 1 h. The images were acquired with a Leica Stellaris 5 confocal microscope (Leica, Mannheim, Germany).

### 2.7. lncRNA Synthesis

For lncRNA synthesis, full-length DNA fragments of lncRNA were inserted into the pCS2+ vector with poly A terminator removed by double digestion of Xho I and Not I enzymes. For PGC-specific overexpression of lncRNAs, the 3′UTR of *nos3* was fused to the 3′ end of the lncRNA in the pCS2+ vector. The recombinant plasmids were linearized with Not I enzyme and transcribed using SP6 mMESSAGE mMACHINE Kit (Invitrogen, Waltham, MA, USA).

### 2.8. Knockdown and Overexpression

Sequence-specific antisense oligonucleotides (ASOs) were synthesized (RiboBio, Guangzhou, China). Different dosages of *lnc196*, *lnc304*, and *lnc172* ASOs and control ASO were injected into 1-cell stage *Tg* (*kop: EGFP-UTR-nanos3*) embryos, respectively. The injected embryos were collected for qPCR analysis and tracing PGC development. For overexpression, 200 pg lncRNAs were injected to 1-cell stage embryos, and embryos injected with 200 pg *rfp* mRNA were used as control. Rescue experiments were performed by co-injection of 200 pg lncRNAs and 10 pM corresponding ASOs into 1-cell stage *Tg* embryos. All the experiments were performed in more than three independent experiments in triplicate. Each independent experiment was performed in triplicate.

### 2.9. PGC Phenotype Observation

The PGC development was examined at 24 hpf under the stereo fluorescence microscope (SMZ800N, Nikon, Kyoto, Japan). The standard for the phenotype of PGC mislocalization was described as in the previous report [26]. The phenotypes were determined by four independent experiments with four groups of embryos (30–50 embryos per group).

### 2.10. Statistics Analysis

The statistics were calculated and analyzed with SPSS version 20. The qPCR results were analyzed by Student’s t-test, and the phenotypes in the different experimental groups were analyzed by one-way ANOVA. *p* < 0.5 was considered a statistically significant difference.

## 3. Results

### 3.1. Identification and Characterization of lncRNA in Zebrafish PGC by scRNA-seq

**Figure 1:**
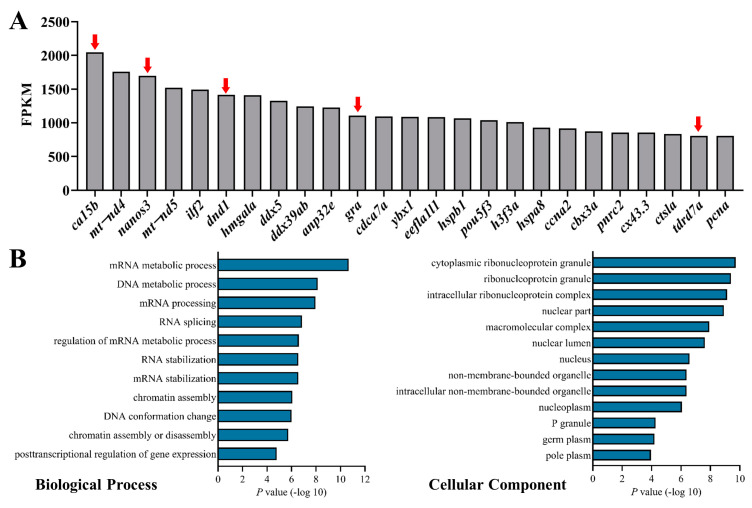
The top 25 genes and GO analysis of top 100 genes in the transcriptome of zebrafish PGCs. (**A**) Top 25 genes in the transcriptome. Arrows indicated the known PGC markers. (**B**) The most enriched biological process (left) and cellular components (right) of the top 100 genes in the transcriptome.

Single PGC was collected with a capillary tube from the embryos of a PGC-specific transgenic line (*Tg kop: egfp−UTR−nos3*) at 5 h post fertilization (hpf) as previously described [45] (Figure S1). The collected PGCs were independently divided into three groups for scRNA-seq by Illumina platform. The transcriptome data obtained 274,016,310 clean reads, which were annotated to 23,099 genes. In the top 25 genes, there were five known PGC marker genes including *ca15b*, *nanos3*, *dnd1*, *gra*, and *tdrd7a* and two mitochondria components, *mt-nd4* and *mt-nd5* (Figure 1A). Gene ontology analysis showed that the top 100 genes were enriched in the components and biological processes that were matched with the intrinsic features of PGC, including cytoplasmic ribonucleoprotein granule, non-membrane-bounded organelle, germ plasm, and the biological process associated with RNA/mRNA metabolism, stabilization, chromatin assembly, and post-transcriptional regulation of gene expression (Figure 1B,C). The top genes and their enriched functional signaling pathways demonstrated that the cells used for scRNA-seq are PGCs.

**Figure 2:**
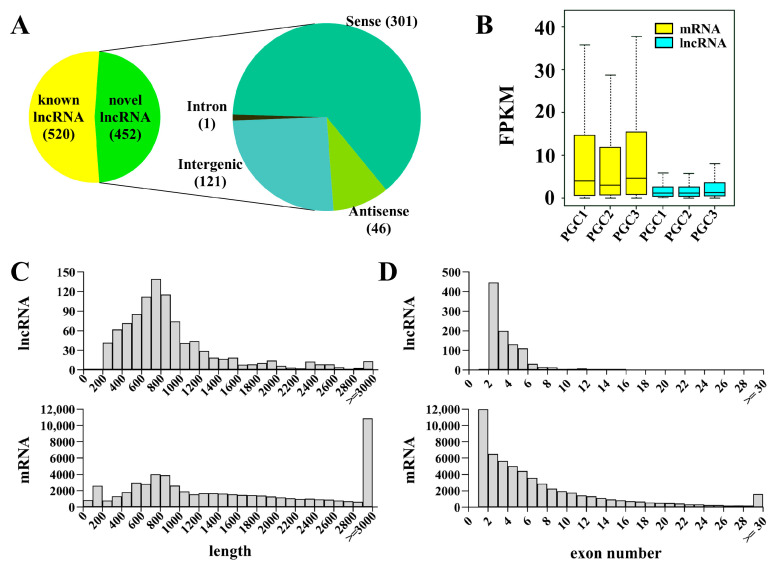
Identification and characterization of lncRNAs in zebrafish PGCs. (**A**) A pie chart showing the lncRNAs and classification of novel lncRNAs. (**B**–**D**) The comparison of FPKM value (**B**), transcript length (**C**), and exon number (**D**) between lncRNAs and mRNAs.

To construct an integrated and stringent set of lncRNA profiles of PGCs, known lncRNAs were obtained by blast to known lncRNA databases and novel lncRNAs were predicted by stepwise computational pipeline as previously described [43]. The resulting obtained 520 known lncRNAs and 452 novel lncRNAs are divided into 4 categories: sense lncRNAs (301, 66.6%), intergenic lncRNAs (121, 26.8%), antisense lncRNAs (46, 10.2%), and intronic lncRNAs (1, 0.2%) (Figure 2A). Compared to mRNAs, lncRNAs have lower expression, are shorter in length, and have fewer exons (Figure 2B–D).

### 3.2. Screening Out lncRNA Candidates Associated with PGC Development in Zebrafish

**Figure 3:**
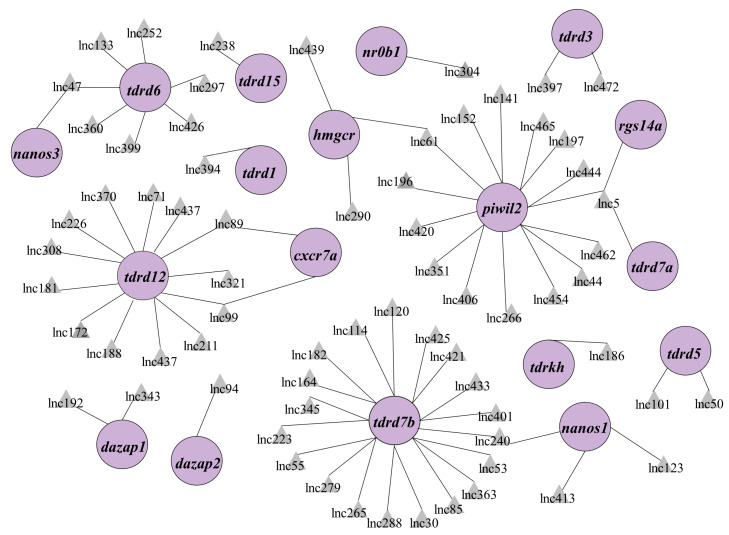
The regulation network of lncRNA-mRNA associated with PGC development. PGC development–associated genes are shown in the purple circles and their regulatory lncRNAs are shown in the grey triangles.

**Figure 4:**
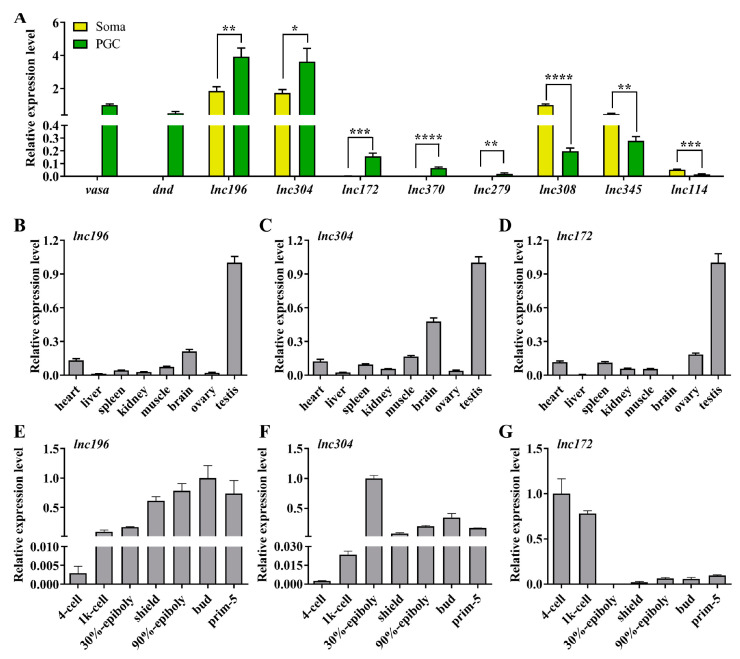
qPCR analysis of top 8 lncRNAs in lncRNA-mRNA network. (**A**) Relative expression of *vasa*, *dnd* and selected lncRNAs in PGC and soma. (**B**–**G**) Tissue distribution and embryonic expression of *lnc196* (**B**,**E**), *lnc304* (**C**,**F**), and *lnc172* (**D**,**G**). * *p* < 0.01, ** *p* < 0.01; *** *p* < 0.001 and **** *p* < 0.0001.

As regulatory transcripts, lncRNAs themselves have limited potential to code protein products; therefore, they usually interact with other coding genes to modulate the biological process. To characterize the role of lncRNA in PGC development, cis- and trans-targets of the novel lncRNAs have been predicted. The results revealed that 147 lncRNAs were potentially targeted to 112 mRNAs, forming 1352 pairs of lncRNA and mRNA (Table S2). To facilitate the search for lncRNA associated with PGC development, we focused on the known important PGC factors within predicted targets and their lncRNAs to construct a regulation network consisting of 69 lncRNAs and 18 known PGC development–associated genes including *tudor domain containing* (*tdrd*) family, *piwi-like RNA-mediated gene silencing 1* (*piwil1*), *chemokine (C-X-C motif) receptor 7 (cxcr7), gamma-glutamyltransferase 1a* (*ggt1*)*, regulator of G protein signaling 14a* (*rgs14a*), and *nanos homolog 1* (*nos1*) (Figure 3, Table S3). From the network, we selected the top nine lncRNAs to analyze their expression in zebrafish PGCs and soma. The qPCR results showed that *lnc196*, *lnc304*, *lnc172*, *lnc370*, *lnc279*, and *lnc181* were PGC-biased, whereas *lnc114*, *lnc308*, and *lnc345* were soma-biased (Figure 4A). Because *lnc196*, *lnc304*, and *lnc172* were the most PGC-enriched lncRNAs, the spatial and temporal expression and localization were further characterized in adult tissues and embryos. In adult tissues, *lnc196*, *lnc304*, and *lnc172* were all enriched in the testes (Figure 4B–D). During embryonic development, *lnc196* existed in the unfertilized egg and increased in tandem with the embryonic development; *lnc304* was expressed from the shield stage; the maternally-expressed *lnc172* quickly decreased and was almost undetectable at 30% epiboly (Figure 4E–G).

**Figure 5:**
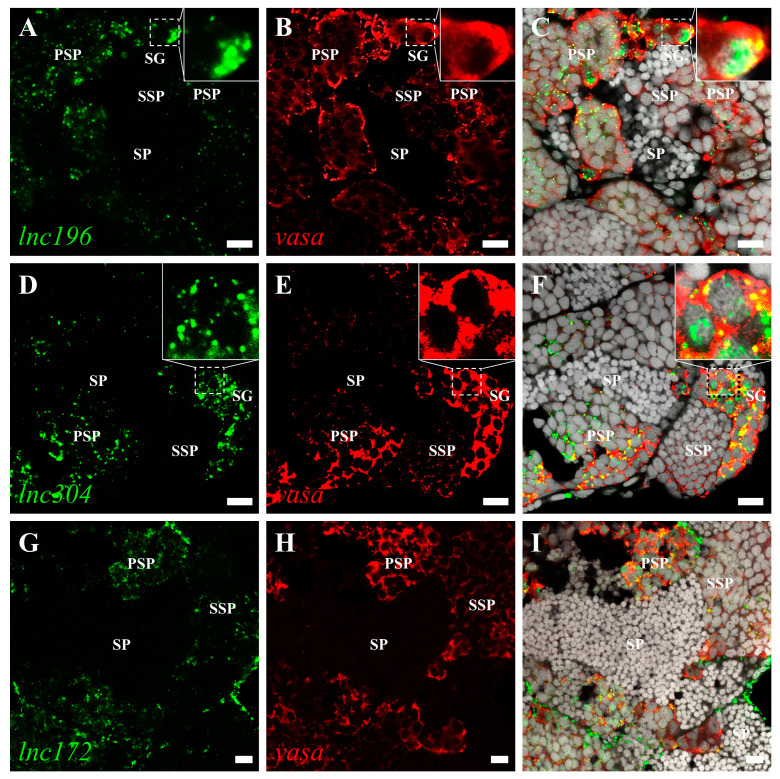
Double color fluorescent analysis of lncRNAs and vasa protein in zebrafish testes. The signals of *lnc196* (**A**), *lnc304* (**D**), and *lnc172* (**G**) are shown in green, and vasa signals (**B**,**E**,**H**) are indicated in red. (**C**,**F**,**I**) The merged images and magnified images are shown in the boxes within panel C and F. SG: spermatogonia, PSP: primary spermatocyte, SSP: secondary spermatocyte and SP: spermatid. Scale bar:10 μm.

**Figure 6:**
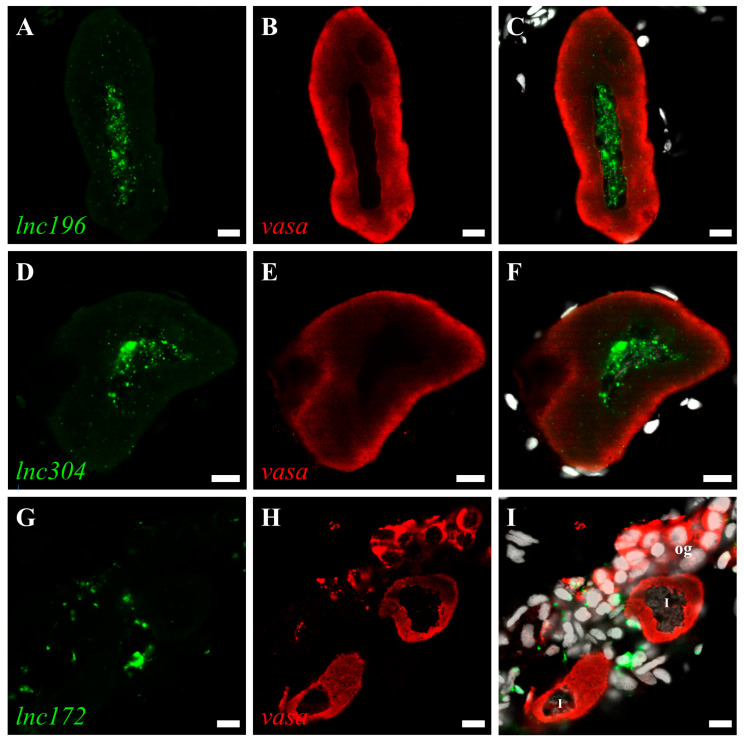
Double color fluorescent analysis of lncRNAs and vasa protein in zebrafish ovary. The signals of lncRNAs *lnc196* (**A**), *lnc304* (**D**) and *lnc172* (**G**) and vasa signals (**B**,**E**,**H**) are shown in green and red, respectively. (**C**,**F**,**I**) Merged images. og: oogonia, I: stage I oocyte. Scale bar:10 μm.

The roles of lncRNA are closely associated with subcellular localization. Therefore, we characterized the subcellular localization of *lnc172*, *lnc196*, and *lnc304* in zebrafish gonads by fluorescent in situ hybridization (FISH). In mature testes, *lnc196* and *lnc304* were localized in the nucleus and cytoplasm of spermatogonia and spermatocytes, and their signals gradually weakened from the spermatogonia over primary spermatocyte to secondary spermatocyte (Figure 5A–F). Notably, both *lnc196* and *lnc304* were partially co-localized with vasa in the cytoplasm of the spermatogonia (the magnified images in panel C&F) (Figure 5). By contrast, *lnc172* was localized in the cytoplasm of the spermatogonia, spermatocyte, and myoid cell (Figure 5G–H). In a mature ovary, *lnc196* and *lnc304* were mainly localized in the nucleus of the pre-vitellogenesis oocyte (Figure 6A–F), and *lnc172* was localized in the cytoplasm of the ovarian somatic cell (Figure 6G–H). The germline- enriched expression patterns suggested that *lnc172*, *lnc196*, and *lnc304* might regulate PGC development.

### 3.3. Disruption of lnc172 and lnc304 Affected PGC Development

**Figure 7:**
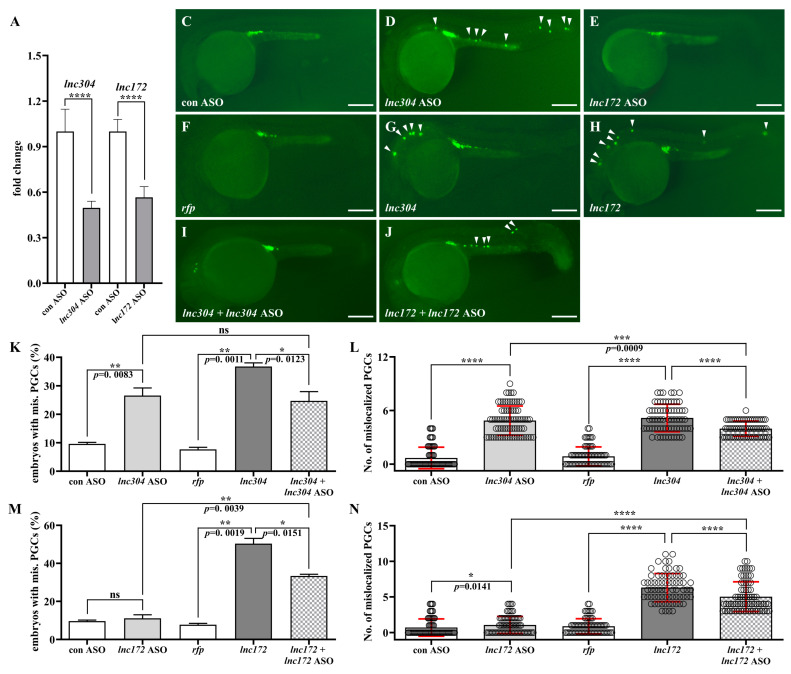
Loss- and gain-of *lnc304* and *lnc172* affected PGC development. (**A**) The fold change in endogenous *lnc304* and *lnc172* in embryos injected with *lnc304* ASO gradients and *lnc172* ASO gradients in comparison with embryo injected with control ASO (con ASO), respectively. (**B–I**) Representative images of the GFP-labelled PGCs in the embryos injected with con ASO (**B**), *lnc304* ASO (**C**), *lnc172* ASO (**D**), *rfp* (**E**), *lnc304* (**F**), *lnc172* (**G**), *lnc304* + *lnc304* ASO (**H**) and *lnc172* + *lnc172* ASO (**I**), respectively. Arrowheads indicate mislocalized PGCs. (**J–M**) The percentage of embryos with mislocalized PGC (**J**,**L**) and the number of mislocalized PGCs in each embryo (**K**,**M**) after injection of different RNAs and ASOs shown in (**B**–**I**) above. The columns represented for mean ± SD. Results were representative of more than three independent experiments in triplicate. *ef1a* and *rpl13a* were used as internal controls to normalize gene expression levels with 2^-ΔΔCt^ method. * *p* < 0.05; ** *p* < 0.01; *** *p* < 0.001, **** *p* < 0.0001. Scale bar, 100 µm.

To investigate the potential role of *lnc172*, *lnc196*, and *lnc304* in PGC development, we performed loss of- and gain-of-function experiments by injection of sequence-specific antisense oligonucleotides (ASO) and lncRNAs, respectively. The qPCR results indicated that injection of ASOs significantly decreased endogenous lncRNAs (Figure 7A and Figure S2A). Next, we examined the knockdown of lncRNAs affected PGC development. Compared to the control ASO-injected embryos, PGC developed normally in *lnc172* or *lnc196*. The ASO- injected embryos are shown at 24 h post fertilization (hpf) in Figure S2. There was significant PGC mislocalization in the *lnc304* ASO-injected embryos (Figure 7B–E and Figure S2B,C,F,G). In the *lnc304* ASO-injected embryos, the percentage of embryos with mislocalized PGC and the average number of mislocalized PGC in each embryo increased to 26.56% and 4.88, respectively (Figure 7K,L). We also investigated whether overexpression of the lncRNAs affected PGC development. Compared to *rfp* mRNA-injected embryos, we observed mislocalized PGCs in the *lnc172*- and *lnc304*-injected embryos (Figure 7F–H) but not in the *lnc196*-injected embryos (Figure S2D–G). The percentage of embryos with mislocalized PGCs and the average number of mislocalized PGCs in each embryo increased to 36.77% and 5.17 and 50.37% and 6.31 in the *lnc304* and *lnc172* injected embryos, respectively (Figure 7K–N). Co-injection of the *lnc304* and *lnc172* ASOs with corresponding lncRNAs significantly decreased the percentage of embryos with mislocalized PGCs and the average number of mislocalized PGCs (Figure 7I–N). These loss of- and gain of-function results indicated that *lnc172* and *lnc304* played important roles in PGC development. 

### 3.4. lnc172 and lnc304 Regulate PGC Development in Different Ways

**Figure 8:**
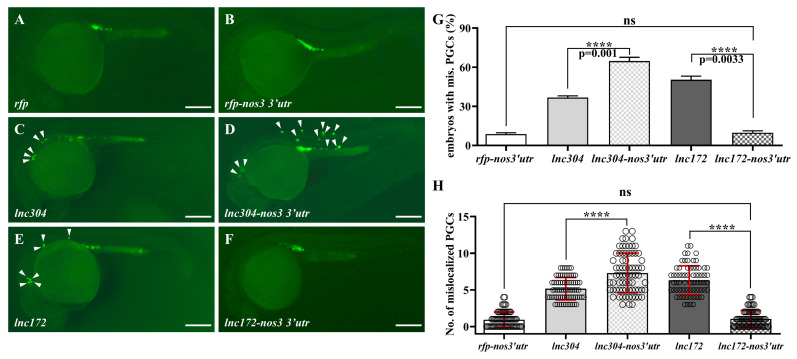
PGC-specific overexpression of *lnc304* affected PGC development. (**A**–**F**) Representative images of the GFP-labelled PGCs in the embryos injected with *rfp* (**A**), *rfp-nos3′utr* (**B**), *lnc304* RNA (**C**), *lnc304-nos3 3′utr* (**D**), *lnc172* RNA (**E**) and *lnc172-nos3 3′utr* (**F**). Arrowheads indicate mislocalized PGCs. (**G**,**H**) The percentage of embryos with mislocalized PGC (G) and the number of mislocalized PGCs (H) in each embryo after injection of different RNAs in (**A–F**). (**H**) The columns represented for mean ± SD. The results were representative of more than three independent experiments in triplicate. **** *p* < 0.0001. Scale bar, 100 µm.

Universal overexpression of *lnc172*- and *lnc304*-mediated developmental abnormalities might be caused by lncRNA overexpression in soma, PGC, or both. To clarify this, we fused the lncRNAs with 3′ untranslated region of *nos3* (the *lnc304-nos3 3′utr* and *lnc172-nos3 3′utr*) that will be specifically overexpressed in the PGCs because of somatic clearance by the miR-430 from gastrulation [46]. Compared to the embryos injected with *lnc304* (Figure 8A–C), the percentage of embryos with mislocalized PGC and the average number of mislocalized PGC in each embryo significantly increased to 64.71% and 7.29 in the *lnc304-nos3 3′utr* injected embryos (Figure 8D,G). By contrast, we found normal PGC development in the *lnc172-nos3-3′utr*-injected embryos (Figure 8E,F,H), suggesting that the developmental defects of PGC in the *lnc172* injected embryos required somatic overexpression of *lnc172*. These results indicate different ways *lnc304* and *lnc172* function in the regulation of PGC development: PGC-specific overexpression of *lnc304* and soma overexpression of *lnc172* disrupted the PGC development.

## 4. Discussion

In the present study, we revealed lncRNA profiles of zebrafish PGC, established the regulation network of lncRNA-mRNA associated with PGC, and found two novel lncRNAs *lnc172* and *lnc304*, which play important roles in PGC development. To the best of our knowledge, it is the first report about the function of lncRNAs in the PGC development of fish.

Zebrafish PGCs specify at ~3 hpf, polarize at ~4.5 hpf, initiate to migrate at ~5 hpf, and reach to the genital ridge and stop migration at 24 hpf [47]. Genome-wide transcriptional silence is required for PGC specification and migration in fruit flies and zebrafish [32,48]. A recent study reports that migratory PGCs at 6 and 11 hpf shared a similar gene expression pattern [41]. In this study, we revealed the lncRNA profiles of zebrafish PGC at 5 hpf when PGCs initiate migration, providing useful information for studying the role of lncRNA in PGC development.

In the lncRNA-mRNA regulation network, there were 12 known RNPs including *piwil2*, *dazap1*/*2*, *tdrd1*/*2*/*3*/*5*/*6*/*7a*/*12*/*15*, and *tdrdkh*. *Piwil*2 interacts with the piRNAs, which is critical for maintaining transposon silence in zebrafish PGC [24]. The germline-specific family TDRDs are indispensable for gametogenesis and genome stability via the piRNA pathway [49,50]. DAZ families are required for PGC migration, as is commitment to germline fate by regulation of RNA stability and translation [23,51,52]. The regulation network suggested that these germline-specific RBPs also interact with lncRNAs. Besides, six lncRNAs were predicted to target with three PGC migration factors including *rgs14a*, *cxcr7a*, and *hmgcr* [10,19,53,54], suggesting that they might be involved in the regulation of PGC migration. From the network, we identified six PGC-based novel lncRNAs including *lnc196*, *lnc304*, and *lnc172*, which were predicted to regulate *piwil2*, *nr0b1*, and *tdrd12*, respectively. However, whether other lncRNAs involved in the regulation network might be associated with PGC development and the interaction among these lncRNAs and PGC- associated genes is a question that should be investigated in the future.

Generally, nucleus-localized lncRNAs are associated with the regulation of chromatin topology and neighboring genes [55,56,57], and cytoplasm-localized lncRNAs act as scaffolds of proteins and RNA to regulate maintenance and decay [58,59]. In this paper, we investigated the subcellular localization of *lnc172*, *lnc196*, and *lnc304* in embryos and gonads and observed strong signals in the gonads (Figure 5 and Figure 6), but we failed to find a specific signal in PGCs (data not shown). We speculated that the reason might that in situ hybridization is not sensitive enough to detect low levels of lncRNA in PGC in comparison with qPCR analysis. In the testes, *lnc196* and *lnc304* were co-localized with vasa in the spermatogonia, and the signals weakened from the spermatogonia to the secondary spermatocyte, which is consistent with the expression pattern of fish germ plasm components [60,61,62]. The nucleus localization of *lnc196* and *lnc304* in male germline and pre-vitellogenic oocyte indicated that they might be associated with chromatin structure and transcriptional regulation. *lnc172* was a highly expressed myoid cell and ovarian somatic cell, which are the gonadal somatic cells that play important role in germline self-renewal, differentiation, and maintenance via sexual hormones synthesis and response [63,64,65]. In summary, the subcellular localization suggests that *lnc172*, *lnc196*, and *lnc304* might play important roles during gametogenesis.

Zebrafish PGCs employ “amoeboid migration” to migrate towards the genital ridge, which is fine-tuned by intrinsic factors and the surrounding niche [5,66]. The onset of PGC migration accompanies the decrease in E-cadherin, whose expression is controlled by Rgs14a, a scaffolding protein in PGCs [19]. The bleb formation and motility of PGC require high myosin-based contractility and cortex properties [67]. Soma-derived chemokine signaling is indispensable for the guiding of PGC to arrive at their specific intermediate and final targets. Disruption of factors involved in chemokine signaling such as *sdf1*, *cxcr4*, and *cxcr7* destroy directional migration, leading to PGCs dispersed in the trunk [68,69,70]. In the present study, we found that knockdown or PGC-specific overexpression of *lnc304* and universal overexpression are probably due to the somatic overexpression of *lnc172*, resulting in PGC scatted in the tail, somite, eyes, and brain (Figure 7 and Figure 8), similar to the phenotypes observed in *cxcr4* morpholino-injected embryos. These results demonstrated that proper expression of lncRNAs in soma and PGC is important for directing PGC migration towards the genital ridge. However, the detail process and mechanism of how *lnc172* and *lnc304* regulate PGC need to be further investigated. 

In summary, the present study provided lncRNA profiles of zebrafish PGC and identified two novel lncRNA critical in directing PGC migration, which is helpful for understanding the role of lncRNA in PGC development.

## Data Availability

All the data in this study are available in the figures and supplemental documents of this paper.

## Supplementary Materials

The following supporting information can be downloaded at: https://www.mdpi.com/article/10.3390/cells12040672/s1, Figure S1: The procedure of PGC isolation for scRNA-seq; Figure S2: PGC development in embryos injected with lnc196 RNA or lnc196 ASO; Table S1: The sequence information of the primers and lncRNAs; Table S2: The predicted cis- and trans-targets of novel lncRNAs; Table S3: The sequence of lncRNA and ensemble ID of corresponding target RNA in network.
