# Supplementary material for: Two Novel lncRNAs Regulate Primordial Germ Cell Development in Zebrafish
Source: Cells. 2023 Feb 20;12(4):672. doi: 10.3390/cells12040672 (PMC9954370; doi:10.3390/cells12040672)
Supplement: Supplementary file 1 [file cells-12-00672-s001.zip › Table S1 The primer information and sequence of lncRNA196, lnc172 and lnc304.pdf]

Description	Primer Name	Primer Sequence (5'-3')
pCS2 + mRNA synthesis	*lnc196*-EcoR1-F	ACCGAATTCATCCCCGGAGCGCCTAGCCTA
	lnc196-Not1-R	CCCGCGGCCGCTTGCAAAAGAAAAAACATTTTATTTGAAAACAT
	lnc304-BamH1-F	CGCGGATCCGCGGCAAACTAGCGAGGGAATC
	lnc304-EcoR1-R	CCGGAATTCCGGTGGTGTTTCCTTGGTCCTC
	lnc172-BamH1-F	CGCGGATCCCATGAACACTCGCTGTCCAG
	lnc172-Xhol-R	CCGCTCGAGTATTTACCACATCACTGCAG
qRT-PCR	*ef1a*-RT-F	GGCTGACTGTGCTGTGCTGATTG
	ef1a-RT-R	CTTGTCGGTGGGACGGCTAGG
	rpl13a-RT-F	TCTGGAGGACTGTAAGAGGTATGC
	rpl13a-RT-R	AGACGCACAATCTTGAGAGCAG
	vasa-RT-F	GAGCATGCAGTGATGTGGAG
	vasa-RT-R	CCGATCACCATGGATGCTTG
	dnd-RT-F	GGCTAAGAAAGTGCTCGTGG
	dnd-RT-R	GCTGGGACGTCATAATGCAG
	lnc196-RT-F	AAGGTGGCGTCAATACTGGA
	lnc196-RT-R	TGGTTCCTGATGCACGGATA
	lnc304-RT-F	AGGGCCTAACAAGTTGAA
	lnc304-RT-R	TCCCAGCCACAGGATGAC
	lnc172-RT-F	CCGAATCCTCATGTCTGCTT
	lnc172-RT-R	TACCACATCACTGCACGACA
	lnc370-RT-F	GCAGCACACAGGAGACTGAA
	lnc370-RT-R	GAGCTGCATGAGCTGAACTG
	lnc279-RT-F	TCATCGTCCAGGAAGTGACA
	lnc279-RT-R	ATGGTTCAGCTTCCTGCTGT
	lnc181-RT-F	CAGATGTGGTGGTGTTGGAG
	lnc181-RT-R	AGAGCTGGGAACAGGACAGA
	lnc114-RT-F	CAGCGTACGGATCTCACAGA
	lnc114-RT-R	AAACGCCAGTGGGTCACTAC
	lnc308-RT-F	GTTGTGATGGAGCTGGACCT
	lnc308-RT-R	CAGAACTGCTGCTGTCTTGC
	lnc345-RT-F	GCACATATTTCAGCCCATCA
	lnc345-RT-R	GGAGGATGTGAAGCTCAGGA
DIG-probe	*lnc196*-F	GGTGGCTGATTAGAGACGGA
	lnc196-R-T7	TAATACGACTCACTATAGCTGTTTTAGTCCTTTGGTCCA
	lnc304-F	CCCCAGAGCAGTCTAACCTT
	lnc304-R-T7	TAATACGACTCACTATAGGTGTTTCCTTGGTCCTCC
	lnc172-F	CATGAACACTCGCTGTCCAG
	lnc172-R-T7	AATACGACTCACTATATATTTACCACATCACTGCACGACACAACAC

>lnc196

ATCCCCGGAGCGCCTAGCCTACTCTGCCCGGATGCTAACGGCAACACAGGACGGTTTC
CATAAGCTCTGGAGAGTTTCTAAAACATATCTGCCACAGTTCTCTATACAATAACACAA
GACCCTGTGTCGGTGGCTGATTAGAGACGGATGGTGTATTTCCAGAGTTGGTGAAATG
ACCAAGCACACCTTCTTGGTCATATGTGGTGATAATAATGAAGTTAAGATTATATTTGGA
CCTGAAACATCTGCTAGACTCCAGGAAAGGTGGCGTCAATACTGGACCTGAAAGGGCT
TAACAAGTTGAATCTGCTGGGAGAGTCAAGTTGTGGATCATCATGAAGATTTTCAAAA
GGCGAGTTTAGATTTCTTGAATGTTTTATTTACTTTAAATGTTCTTTTAAATATATCATAG
AATTTGATTGCAATTTGTTTTCAAACATTTTTCTTAGTCATGCTGAAGTCTTGAAGAGTT
CTTAGATGAGCATCACCCTATTTCCGTGCATCAGGAACCAGCAGTCGAGGGATCAGCA
GCTACTACATCGTCATGGATAAAGGTCACCCTGTGGCTGGGAAGCACTTCATTCACAGG
ACATGTTTGATGAGATTTCCTAGCTCCAGTTTGTTTTCACACAAGTTTACAGCATTTGTA
CCTTCATTTAAACTGCTGTGTTTGATATGGAGGACCAAGGAAAAACACCAGAAGTGAA
AGATTTGCACGCCAAGTTGTTTAAAATGTAATTTCAGAAGAAAACTATGCGCTCAGAA
GAAAACTATGAGTGACGATACTGTATATTGAACTTTCAAATTGCTCTGATGAAACATAA
AGTTTGGACCAAAGGACTAAAACAGCCATTTTGAGCACTGGTCATACATATTTTGTTGA
TACTGTCTGTACTAATAAAACATTTAAAGCATATTTGACATTGTTGCATTTTAAAAACTG
AATGAATTGTTGAAGTGGTGAATGTTTTCAAATAAAATGTTTTTTCTTTTGCAA

>lnc304

GCAAACTAGCGAGGGAATCCCCTGAGCAGCCTAGCCTACTCTGCCCGGATGCTAACGG
CAACACAGGACGGTTTCCATAAGCTCTGGAGAGTTTCTAAAACATGTCTGCCACAGTT
CTCTATACAATAACACAAGACCCTGTGACGGTGGCTGATTAGAGACGGATGGTGTATTT
CCAGAGTTGGTGAAATGACCAAGCACACCTTCTTGGTCATATGTGGTGATAATAATGAA
GATTCTATTTTGACCTGAAACATCTGCTGGACTCCAGGAAAGGTGGCGTCAATACTGG
ACCTGAAAGGGCCTAACAAGTTGAATCTGCTGGGAGAGTCAAGTTGTGGATCATCATG
TAGATTTTCAAAAGTAATGCTGGAGTCTTGAAGAGTTCTCAGATGAGCATCACCCTATA
TCCGTGCATCAGGAACCACCAGATGAGCGATCAGCAGCTACTACATCGTCATGGATAA
AAAGGTCATCCTGTGGCCGGGAAGCACTTCATTCACAGGACATGCTTGATGAAATTTC
CTAGCTCCAGTTTGTTTTCACATAAGTTTACAGCATTTATACCTTCATTTAAACTGCTGT
GTTTGATGTGGAGGACCAAGGAAACACCAGAAGTGAAAGATTTGCACGCCAAGTTGT
TTAAAATGTAATTTCAGAAGAAAACTATGCGTTCAGAAGAAAACTATGAGTGACGATAT
ATTGAACTTTCAAATTGCTCTGATGAAACATCAAGTTTGGACCAAAGGACTGAAACAG
CTTTTTTGAGCACTTGTCGTACATACTTTGTTGATACTGTCTGTACTAATAAAACATTTA
AAGCTAAAAAAAAAAAAAAAAGTACTCTGCGTTGATACCACTGCTT

>lnc172

CATGAACACTCGCTGTCCAGCCTGGTGTGACCGAATCCTCATGTCTGCTTCGGCCAAA
GACCTGATCGGCAAGCCGGAGAACGAGGACAAATCAATAACATACGACAACATCGGA
GCCAACGTGTGTATGGGAGACCACAAGCCGGTCTACTTGTTCTTTCGGTTGACCACAG
GTCCAGGTAAACCTAATGCAAATAAGCACAAGTGTTGTGTCGTGCAGTGATGTGGTAA
ATA
