# Supplementary figures and images for: Two Novel lncRNAs Regulate Primordial Germ Cell Development in Zebrafish
Source: Cells. 2023 Feb 20;12(4):672. doi: 10.3390/cells12040672 (PMC9954370; doi:10.3390/cells12040672)

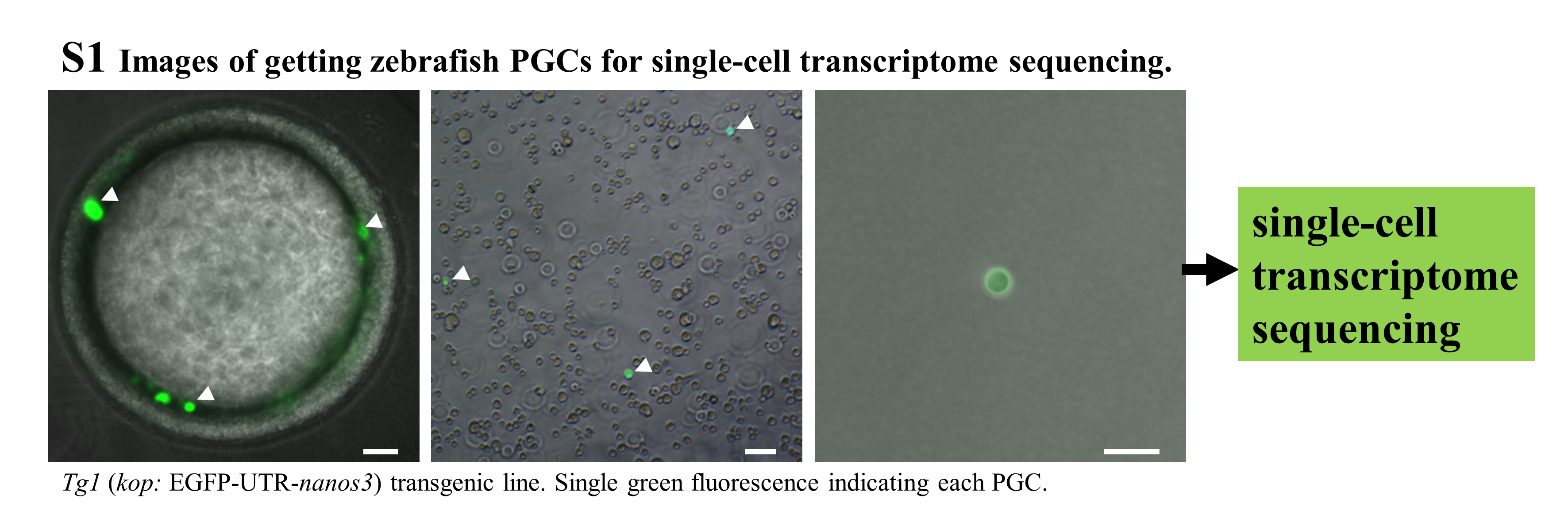

Supplement: Supplementary file 1 [file cells-12-00672-s001.zip › Figure S1.tif]

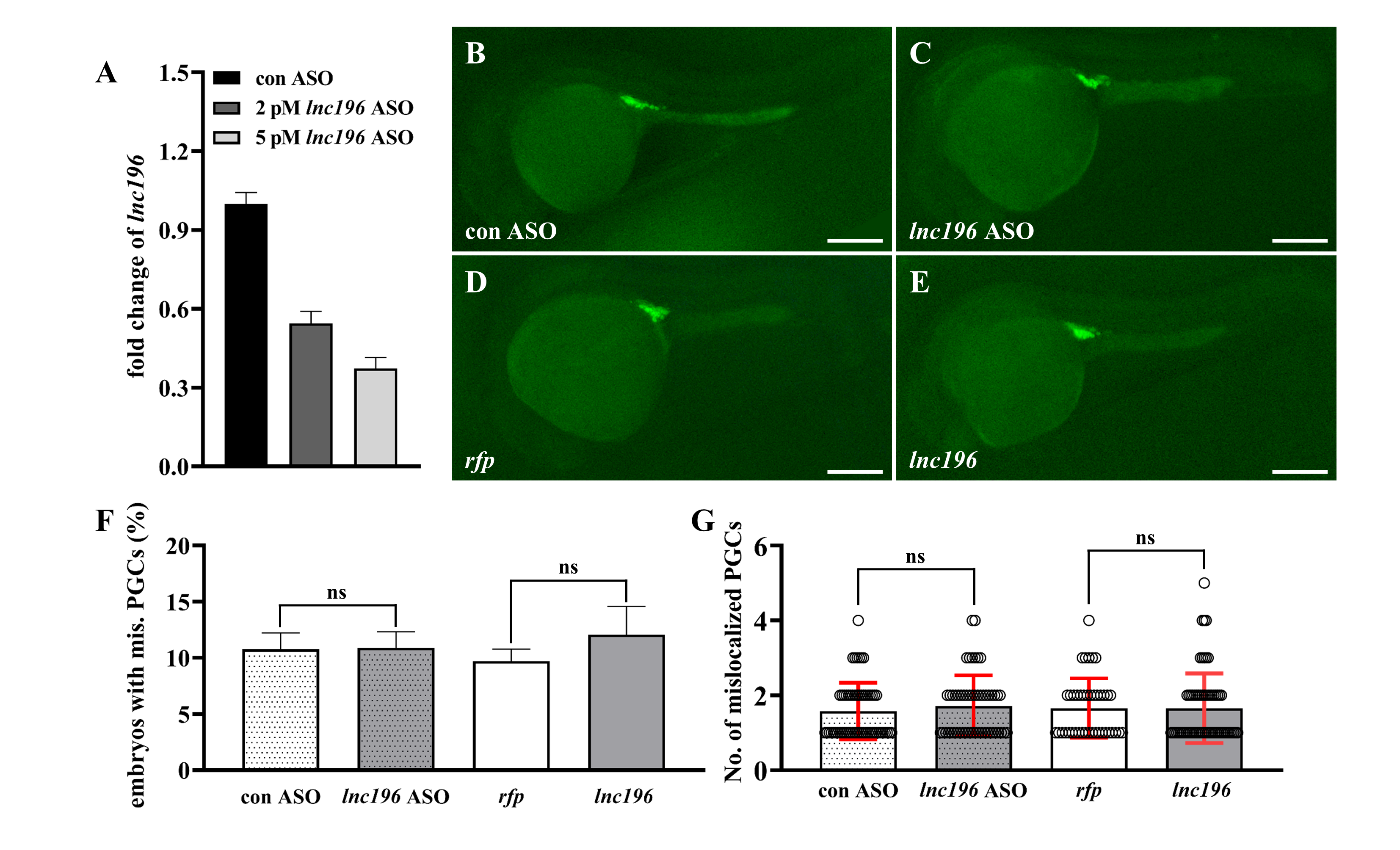

Supplement: Supplementary file 1 [file cells-12-00672-s001.zip › Figure S2.tif]
